# Recent Progress on Multi-Component Reactions Involving Nucleophile, Arynes and CO_2_

**DOI:** 10.3390/molecules29133152

**Published:** 2024-07-02

**Authors:** Shaoxuan Gong, Xiumei Xie, Hongxia Sun, Yuting Liu, Junjie Li, Zhen Zhang

**Affiliations:** 1 Engineering Research Center of Sichuan-Tibet Traditional Medicinal Plant & School of Pharmacy, Chengdu University, Chengdu 610106, China; gongshaoxuan@stu.cdu.edu.cn (S.G.); 13281133352@163.com (X.X.); 13320964726@163.com (H.S.); lyuting398@gmail.com (Y.L.); 18685820575@163.com (J.L.); 2 School of Chemistry and Chemical Engineering, Shanxi University, Taiyuan 030006, China

**Keywords:** carbon dioxide (CO_2_), aryne, multi-component reactions, *ortho*-substituted benzoic acids

## Abstract

Carbon dioxide (CO_2_) is a non-toxic, abundant and recoverable source of carbon monoxide. Despite its thermodynamically stable and kinetically inert nature, research on CO_2_ utilisation is ongoing. CO_2_-based aryne reactions, crucial for synthesising *ortho*-substituted benzoic acids and their cyclisation products, have garnered significant attention, and multi-component reactions (MCRs) involving CO_2_, aryne and nucleophilic reagents have been extensively studied. This review highlights recent advancements in CO_2_ capture reactions utilising phenylalkyne reactive intermediates. Mechanistic insights into these reactions are provided together with prospects for further development in this field.

## 1. Introduction

Carbon dioxide (CO_2_) provides a non-toxic, abundant and recyclable C1 source and has broad application prospects in organic synthesis. However, the approach by which CO_2_ can be activated and utilised remains challenging due to its thermodynamic stability and kinetic inertness [1,2]. During the past few decades, various organic transformations of CO_2_ have been developed [3,4,5,6,7]. Examples of metal-catalyzed [8], photocatalyzed [9] and electrocatalyzed [10,11,12] effective promotion of CO_2_ conversion have been widely reported.

Arynes are highly reactive organic intermediates possessing a triple bond within a molecule intermediate between a double bond and a triple bond. One π-bond belongs to the aromatic system, whereas the other π-bond is formed by the lateral overlap of two sp^2^ orbitals in the plane of the benzene ring [13,14]. The ring strain induced by the formal triple bond on a six-membered ring results in the lowering of its LUMO (lowest unoccupied molecular orbital), leading to a small energy gap between the HOMO (highest occupied molecular orbital) and LUMO, making arynes excellent electrophilic reagents [15,16,17]. Kobayashi’s group [18] first reported 2-(trimethylsilyl)phenyl trifluoromethanesulfonate as a precursor for phenylalkynes in 1983. Under the action of fluoride ions, the trimethylsilyl group dissociates, resulting in the formation of phenylalkyne precursors.

**Figure 1:**
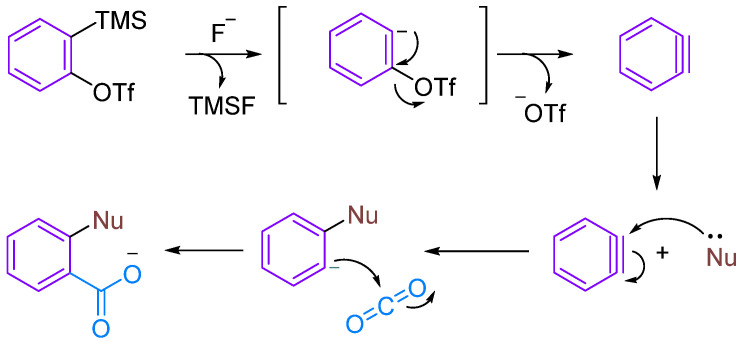
The reaction between Kobayashi benzyne precursor and CO_2_.

Although CO_2_ is thermodynamically stable and kinetically inert, it exhibits certain electrophilicity due to the electron-withdrawing action of oxygen atoms. Therefore, aryne can undergo MCRs under certain conditions, such as when nucleophilic reagents attack to form amphipathic ions that can trap CO_2_ (Figure 1). In this review, we summarise the MCRs of aromatic alkynes with CO_2_ in combination with nucleophilic reagents, discuss the reaction mechanisms underlying this class of reactions and provide an outlook on future developments.

## 2. Nucleophilic Addition Reactions of Aromatic Alkynes Involving CO_2_

### 2.1. Nucleophilic Addition Reactions of Aromatic Alkynes with N-Compounds Involving CO_2_

**Figure 2:**
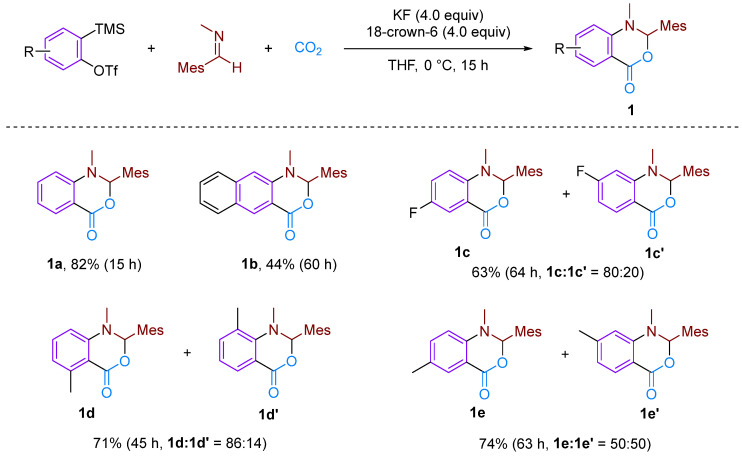
CO_2_-involved three-component coupling reaction between arynes and *N*-(2,4,6-trimethylbenzylidene)methylamine.

**Figure 3:**
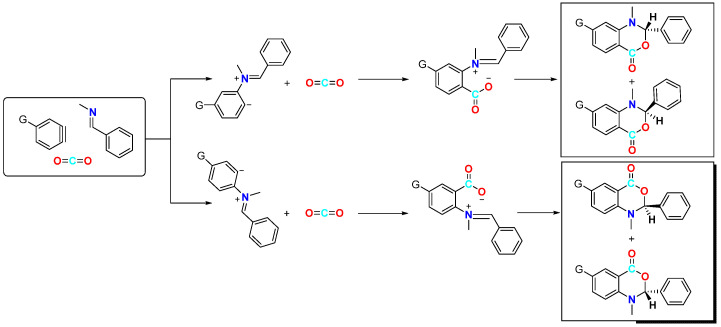
Mechanistic studies of CO_2_-involved three-component coupling reaction between arynes and *N*-(2,4,6-trimethylbenzylidene)methylamine.

In 2006, Hiroto Yoshida et al. first reported the CO_2_-involved nucleophilic cyclisation of three-component aromatic alkynes (Figure 2) for synthesising six-membered heterocyclic benzoxazinones [19]. They observed that the reaction of a newly prepared aryne with *N*-(2,4,6-trimethylbenzylidene)-methylamine under a CO_2_ atmosphere formed a three-component coupling product, benzoxazinone, with a high yield (82%). This reaction was found to occur more efficiently with electron-rich and neutral imines, resulting in higher yields of the coupling product. However, the reaction failed to occur with less nucleophilic imines and imines with larger spatial steric hindrance such as *N*-*t*-Bu and *N*-Ph imine. The reaction mechanism involves attacking the imine on the phenyl alkyne intermediate, resulting in the formation of an amphiphilic intermediate. Subsequently, CO_2_ undergoes electrophilic addition with this amphiphilic anion, forming a carboxylate anion, which ultimately yields the target product. In addition to simple benzene, other substituted aromatics are suitable for this reaction; furthermore, a certain regioselectivity is observed for asymmetrically substituted aromatics. This regioselectivity is attributed to the electronic and spatial effects caused by substituent groups on the arylalkane. Sabet-Sarvestani’s group conducted further mechanistic studies of this reaction [20] and suggested that the reaction is most likely initiated by the nucleophilic attack of the imine (Figure 3).

**Figure 4:**
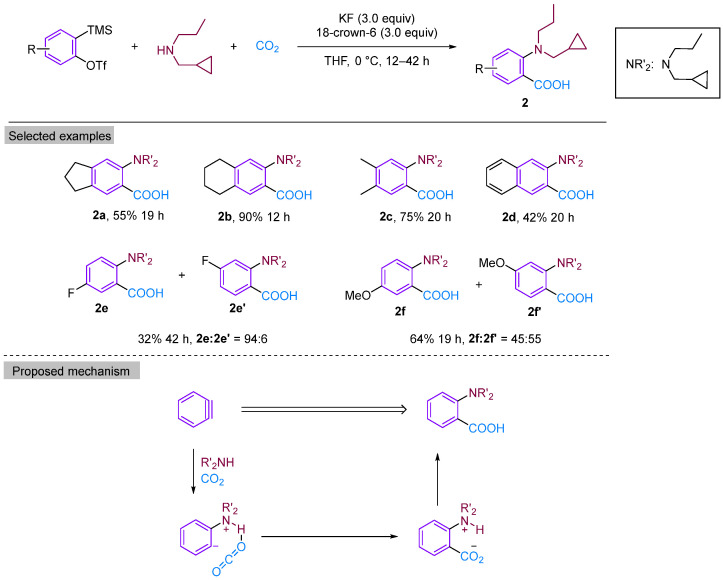
CO_2_-involved three-component coupling reaction between arynes and dipropylamine.

In 2008, Yoshida’s group reported a three-component coupling reaction using a neutral nucleophilic reagent instead of an imine as the substrate [21]. This reaction resulted in the formation of dipropylamine with benzyne to form *ortho*-aminobenzoic acid derivatives under a CO_2_ atmosphere (Figure 4). They explored a range of secondary amine substrates and obtained moderate to high yields of neighbouring amino acids, with the exception of tetrandrine. Secondary amines were found to possess greater stability than imines. Additionally, the use of secondary amines allows for increased structural diversity, leading to a significant expansion in the scope of aromatic ring substrates. According to the proposed reaction mechanism, the electrophilic attack by a secondary amine on phenylalkynes generates amphipathic ions, which are subsequently captured by CO_2_ to produce *o*-aminobenzoic acid through proton migration. The high selectivity of the three-component coupling reaction relative to other amine arylation reactions may be attributed to the formation of intramolecular hydrogen bonds between amine-H and CO_2_, which considerably enhances the electrophilicity of CO_2_. Furthermore, the Lewis basicity of nitrogen effectively inhibits the competition between hydrogen protons and CO_2_, thereby avoiding the formation of protonated products.

**Figure 5:**
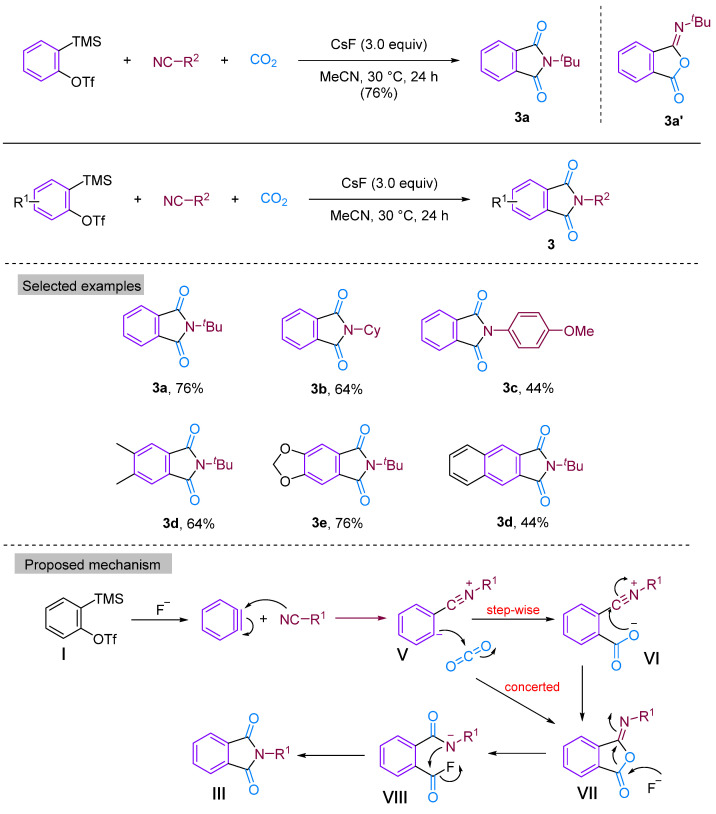
CO_2_-involved three-component coupling reaction between arynes and isocyanides (Kaicharla’s group).

**Figure 6:**
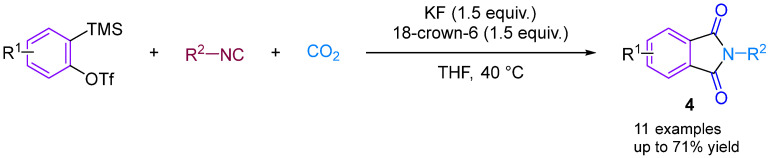
CO_2_-involved three-component coupling reaction between arynes and isocyanides (Yi’s group).

In 2014, Kaicharla’s group reported a CO_2_-involved three-component coupling reaction between phenylalkynes and isocyanides, resulting in the production of *N*-substituted phthalimides [22]. Although they expected an iminoisobenzofuranone derivative **3a** due to the interception of the 1:1 adduct between aryne and isocyanide with CO_2_, a different final product was formed, namely *N*-tert-butylphthalimide **3a′**, but still achieved a high yield of 76% (Figure 5). The reaction proceeds by the nucleophilic addition of isocyanide to the aryl hydrocarbon formed in situ from precursor **I**, leading to the generation of 1,3-amphiphilic intermediate **V**. This nucleophilic aryl anionic intermediate **V** can then progressively combine with the electrophilic carbonyl group of CO_2_ to form the amphiphilic ion **VI**, which subsequently closes the ring to generate the iminoisobenzofuranone derivative **VII**. However, the aryl anion **V** and CO_2_ can also undergo addition and cyclisation to form **VII** via a synergistic reaction, with the subsequent fluorination-induced ring-opening of **VII** generating the acidic fluorine intermediate **VIII**, which is further cyclised to give the phthalic acid imine derivative **III** (Figure 5). Previous studies have documented and elucidated the rearrangement of isoimine **VII** to imine **III** and a fluoride ion-induced rearrangement of isoimine **VII** to phthalimide **III**, which further confirms the authors’ claim [23,24]. In 2015, Yi’s group [25] also obtained phthalimide derivatives (Figure 6) under different conditions, but these derivatives differed from those produced by Kaicharla’s group in terms of their substrate suitability.

**Figure 7:**
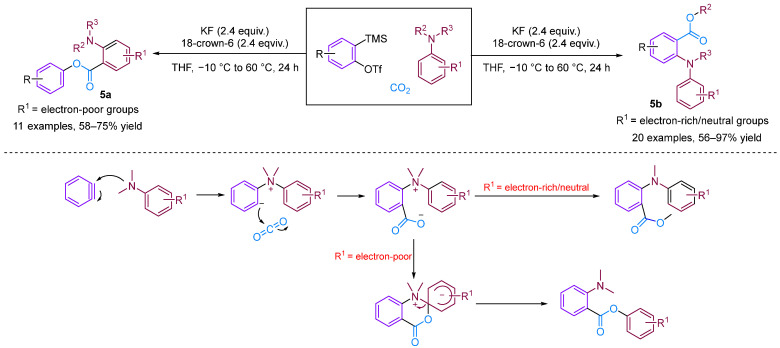
CO_2_-involved three-component coupling reaction between arynes and aromatic tertiary amines.

The Bhojgude group reported transition-metal-free MCC involving arynes [26], aromatic tertiary amines and CO_2_ (Figure 7). Interestingly, this reaction exhibits switchable selectivity depending on the electronic nature of the aromatic amines used. When using amines bearing electron-releasing/neutral groups as the nucleophilic trigger, the reaction afforded 2-arylamino benzoates via nitrogen to oxygen alkyl group migration. However, using electron-deficient amines in the reaction led to the production of 2-aminoaryl benzoates via the aryl-to-aryl amino group migration, resembling a Smiles rearrangement [27].

**Figure 8:**
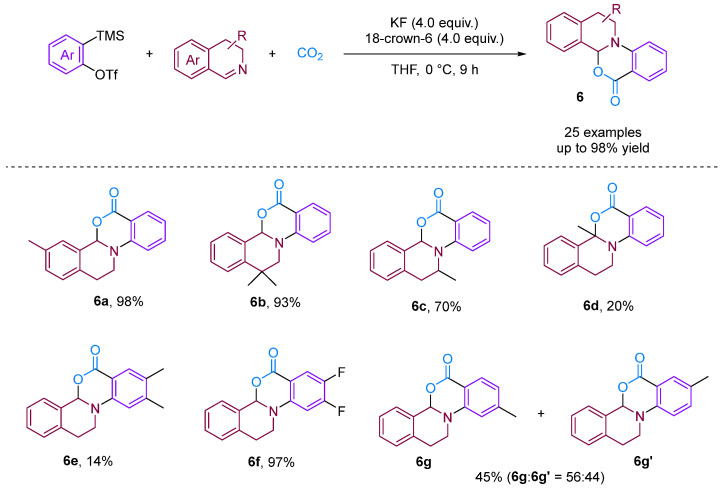
CO_2_-involved three-component coupling reaction between arynes and 3,4-dihydroisoquinolines.

**Figure 9:**
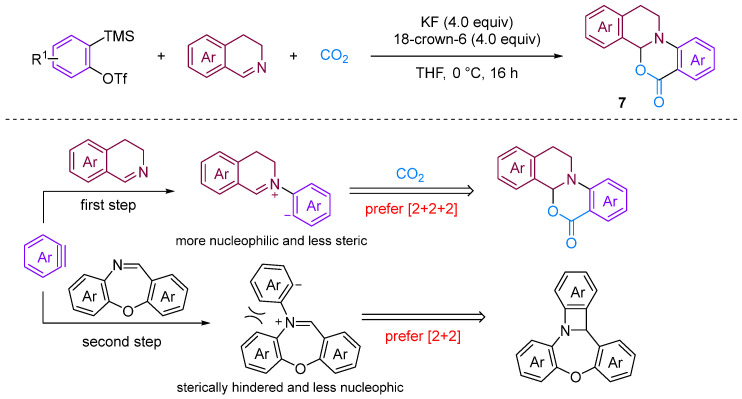
The steric and nucleophilic factors controlled the annuloselectivity.

Chen and Xu [28] developed a formal [2 + 2 + 2] annulation of 3,4-dihydroisoquinolines, CO_2_ and arynes for the efficient synthesis of tetrahydroisoquinoline-fused polycyclic heterocycles (Figure 8). Their method demonstrated the advantages of convergent synthesis and the utilisation of CO_2_ and readily available starting materials; furthermore, this transformation has been successfully scaled up to the gram scale and the resulting annuladducts can be converted into a series of valuable azapolyheterocyclic derivatives. The generation of electron-rich dihydroisoquinolines was found to have superior reactivity. Notably, the transformation efficiency of electron-deficient 3-methoxy benzyne was significantly higher than that of electron-rich 3-methyl benzyne, reflecting the special regioselectivity of the reaction. The observed preference for reactions with electron-rich dihydroisoquinolines and electron-deficient aryne suggests that the rate-limiting step is likely the nucleophilic reaction between imines and benzynes. Notably, when employing dibenzo [1,4]oxazepines as the reactant for the cyclisation reaction with aryne in the presence of CO_2_, [2 + 2], annulation was obtained; this differs remarkably from a previous work in which aryne and CO_2_ with 3,4-dihydroisoquinolines, a representative of *N*-alkyl cyclic imines, was found to deliver exclusively [2 + 2 + 2] annulation products [29]. In [2 + 2] annulation, the Hammett analyses revealed that the nucleophilic attack by imines on benzynes (the first step, negative ρ) and attack by phenyl anions on iminium species (the second step, positive ρ) influence the reaction rate (Figure 9). Based on the incentive verification experiment, the author suggests that the steric and nucleophilic factors controlled the annuloselectivity. Reactivity analysis revealed that the electron-rich and -deficient steric *N*-alkyl cyclic imines and electron-deficient benzynes favour [2 + 2 + 2] annulation, while the electron-deficient and bulky *N*-aryl cyclic imines prefer [2 + 2] annulation [28].

**Figure 10:**
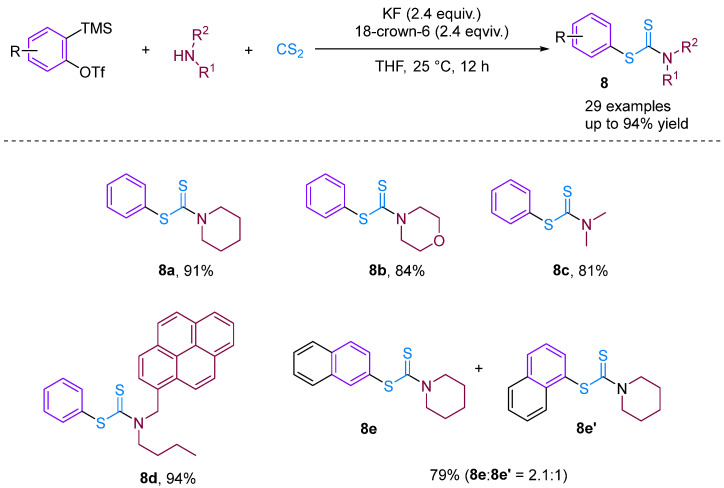
CS_2_-involved three-component coupling reaction between arynes and aliphatic amines.

Recently, based on previous work, Bhojgude, Jindal and co-workers reported three-component coupling involving arynes [30], aliphatic amines and CS_2_ leading to the formation of *S*-aryl dithiocarbamates in high yield (Figure 10). Contrary to known aryne MCC involving amines and CO_2_, the present reaction proceeds via the initial addition of amines to CS_2_ followed by trapping with arynes to obtain the desired products. Detailed experimental and density functional theory studies have demonstrated the addition mechanism involved. Moreover, using 3-triflyloxybenzyne, a four-component coupling with the incorporation of THF was also observed.

### 2.2. Nucleophilic Addition Reactions of Aromatic Alkynes with O-Containing Compounds Involving CO_2_

**Figure 11:**
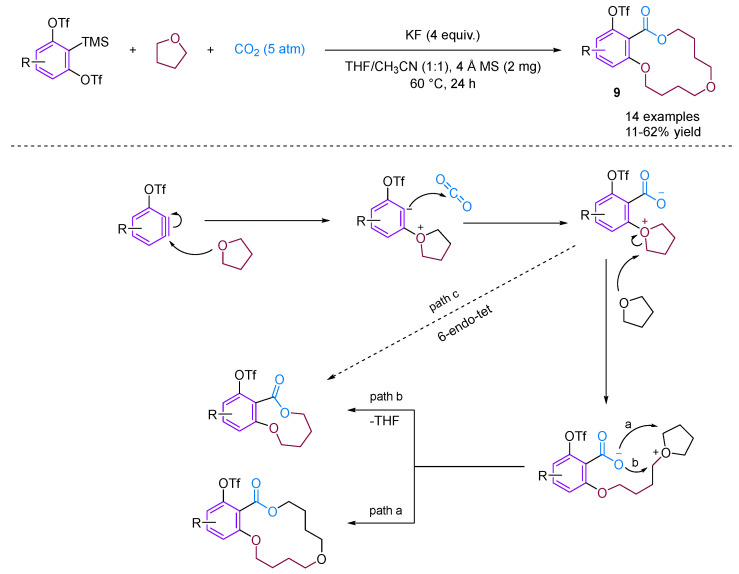
CO_2_-involved three-component coupling reaction between arynes and tetrahydrofuran.

Recently, Qi, Jiang and their team developed a straightforward method for synthesizing a variety of structurally intriguing and valuable 14-membered macrocyclic lactones [31]. This is achieved through a carboxylative macrocyclization of 3-triflyloxybenzynes with CO_2_ and THF, while the novel [2 + 2 + 5 + 5] coupling reaction proceeds under transition metal-free conditions, forming one new C–C bond and three new C–O bonds in a single step (Figure 11).

### 2.3. Nucleophilic Addition Reactions of Aromatic Alkynes with P-Containing Compounds Involving CO_2_

**Figure 12:**
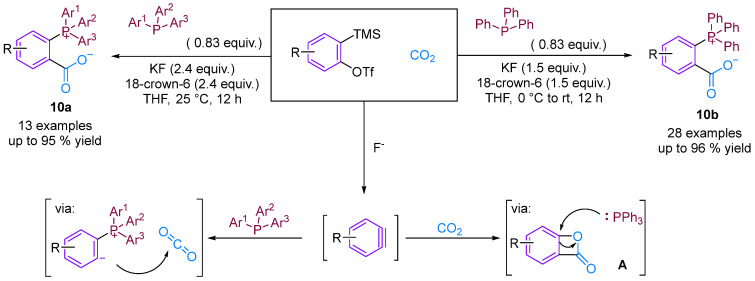
CO_2_-involved three-component coupling reaction between arynes and phosphines.

Considering the potential application of phosphonium salts as ligands, reagents, ionic liquids and organo-catalysts in organic synthesis, zwitterionic phosphonium benzoates developed using the aryne strategy are likely to have important applications [32,33]. Bhojgude, Roy and co-workers reported the triggering of transition metal-free aryne three-component coupling by phosphines using CO_2_ as the third component [34]. The reaction afforded zwitterionic phosphonium benzoates under mild and facile simple conditions instead of the envisioned benzooxaphosphol-3(1*H*)-ones. Mechanistically, in most cases, the initial phosphine–aryne zwitterion was trapped by CO_2_ to form carboxylates in high yield. He, Cai and colleagues prepared the corresponding zwitterionic phosphonium benzoates using a similar method [35]. However, He’s group views the reaction mechanism differently, proposing that the reaction occurs through two mechanisms. The first is similar to that reported by Bhojgude, and the second is via the [2 + 2] cycloaddition of benzyne and CO_2_ to generate intermediate **A**, which subsequently undergoes a ring-opening reaction with triphenylphosphine to produce the final product. Control experiments indicate the possibility of forming intermediate **A** (Figure 12).

### 2.4. Nucleophilic Addition Reactions of Aromatic Alkynes with Halogens Involving CO_2_

**Figure 13:**
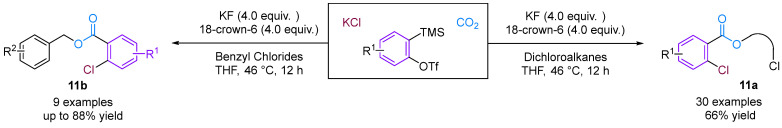
CO_2_-involved 3P-4CR between KCl, arynes and chloroalkanes.

**Figure 14:**
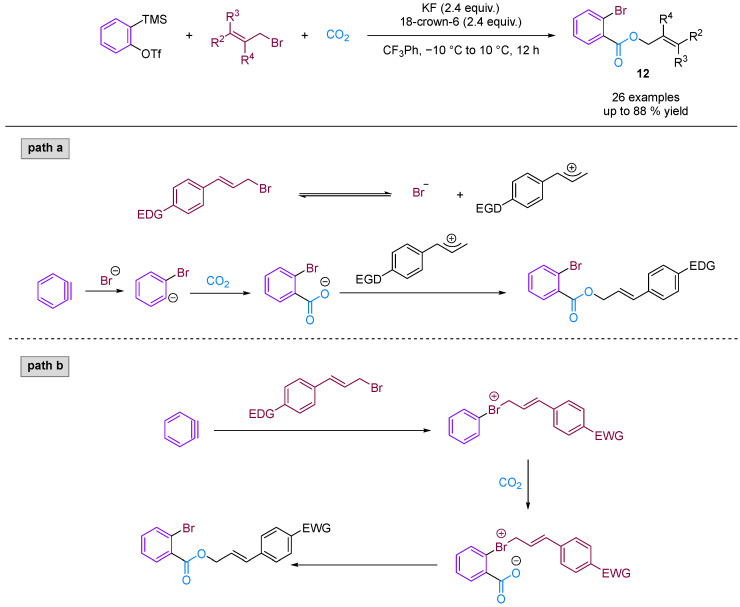
CO_2_-involved three-component coupling reaction between arynes and allyl bromides.

Jiang and co-workers [36] developed a novel three-phase four-component coupling reaction (3P-4CR) using KCl, arynes, chloroalkanes and CO_2_, thereby providing a facile method for synthesising different types of *o*-chloro benzoates (Figure 13). The reaction involves the formation of three different new C–Cl, C–C and C–O bonds in a one-pot fashion. In addition, the chloro and ester groups of the products can be alternatively utilised for diverse transformations. Building on this innovative work, Jiang and colleagues [37] developed an unprecedented three-component coupling involving arynes, allyl bromides and CO_2_, providing efficient and facile access to structurally diverse *ortho*-brominated aryl esters (Figure 14). Distinct from their conventional role as electrophiles in organic synthesis, organic bromides serve as nucleophiles in this reaction, affording a new approach to MCRs involving aryne intermediates. Additionally, the Hammett analyses suggested that two reaction mechanisms were present (path a and path b), depending on the electronic nature of the cinnamyl bromides used in the reaction.

### 2.5. Nucleophilic Addition Reactions of Aromatic Alkynes with Terminal Alkynes Involving CO_2_

**Figure 15:**
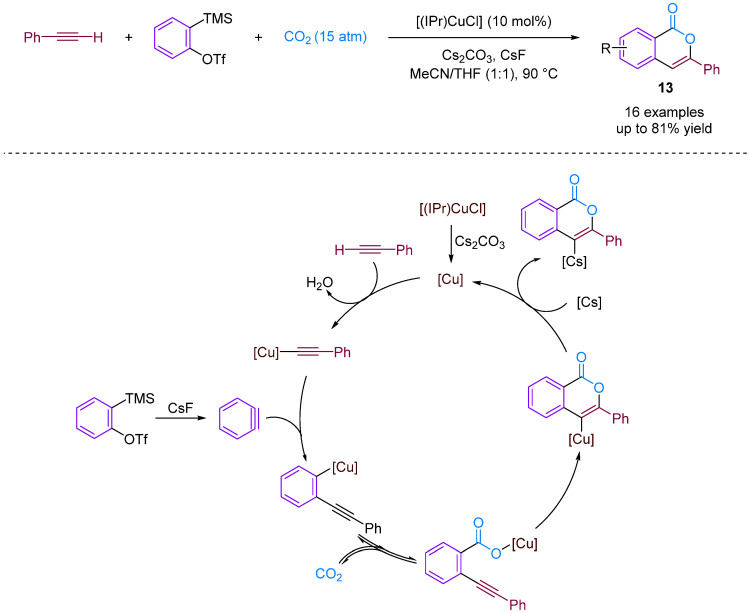
CO_2_-involved three-component coupling reaction between arynes and terminal alkynes catalysed by an NHC–copper complex.

Kobayashi and colleagues (2014) presented the synthesis of isocoumarins using the three-component couplings of arynes, terminal alkynes and CO_2_ catalysed by an NHC–copper complex [38]. Isocoumarins are important lactones found in natural products and exhibit various biological activities. Moreover, new synthetic strategies are being developed to prepare these materials efficiently, such as the intramolecular cyclisation of *ortho*-alkynylbenzoic acid derivatives; however, this requires a multistep synthetic route [39]. A copper-catalysed carboxylation reaction of terminal alkynes with CO_2_ successfully produced isocoumarins. *N*-heterocyclic carbene–copper complexes were found to facilitate multiple transformations in a multi-component reaction. The reaction involved the generation of copper carboxylate and isocoumarins. Furthermore, the synthetic utility of the reaction was demonstrated at the gram scale. The reaction mechanism involves the formation of reactive intermediates leading to desired products (Figure 15).

## 3. Conclusions

CO_2_-involved aryne reactions typically proceed under very mild conditions, requiring minimal strong bases and often relatively low reaction temperatures. This gentle conversion of carbon dioxide, which is a relatively stable compound, is highly significant for translating into carbonyl compounds. In addition, employing various nucleophilic reagents renders it convenient to obtain benzoic acids or cyclisation products with varying substitution patterns, playing a crucial role in pharmaceutical and material synthesis.

In terms of substrate selection, despite the considerable progress, few nucleophilic reagents are currently available for attack, most of which are *N*-containing compounds or their derivatives. The reason for this is twofold. On the one hand, nitrogen atoms possess strong nucleophilicity, allowing them to rapidly attack in situ benzyne intermediates and proceed with subsequent reactions. A more critical factor is that common nucleophilic reagents (such as alcohols and phenols) generate protons in situ during the reaction, which severely hinders the further involvement of carbon dioxide in the reaction and may lead to the formation of large amounts of protonated by-products if not handled appropriately. However, *N*-containing compounds (such as amine compounds) can, to some extent, bind the formed protons due to their Lewis basicity, thereby affording the target product in higher yield, as demonstrated in the reaction shown in Figure 4. Therefore, effectively trapping the generated protons in the reaction system is crucial for the success of similar reactions. Furthermore, the efficient implementation of such transformations can be achieved through substrate design to facilitate ring-closing reactions after addition.

In terms of mechanistic studies, computational evidence has demonstrated that amphipathic ions generated in the nucleophilic attack of aryne by nucleophilic reagents serve as effective CO_2_ trappers. However, mechanistic studies on cycloaddition reactions involving CO_2_ remain relatively underexplored. Furthermore, achieving regioselective control over aryl ring substitution substrates is crucial. Moreover, various reactive intermediates have garnered significant interest for CO_2_ activation and should form the focus of future studies.

Overall, CO_2_-involved aryne reactions have many promising applications for synthesising diverse and meaningful molecular backbones, provided that challenges such as substrate selection, mechanistic understanding and regioselective control are addressed effectively.

## Data Availability

Not applicable.
